# Formulation of Sustained Release Hydrophilic Matrix Tablets of Tolcapone with the Application of Sedem Diagram: Influence of Tolcapone’s Particle Size on Sustained Release

**DOI:** 10.3390/pharmaceutics12070674

**Published:** 2020-07-17

**Authors:** Anna Nardi-Ricart, Isaac Nofrerias-Roig, Marc Suñé-Pou, Pilar Pérez-Lozano, Montse Miñarro-Carmona, Encarna García-Montoya, Josep R. Ticó-Grau, Raul Insa Boronat, Josep M. Suñé-Negre

**Affiliations:** 1 Pharmaceutical Technology and Physico-Chemical Department, Universitat de Barcelona, Av. Joan XXIII, 27-31, 08028 Barcelona, Spain; annanardi@ub.edu (A.N.-R.); isaac.roig@gmail.com (I.N.-R.); msunepou@gmail.com (M.S.-P.); perezlo@ub.edu (P.P.-L.); minarromontse@ub.edu (M.M.-C.); jrtico@ub.edu (J.R.T.-G.); jmsune@ub.edu (J.M.S.-N.); 2 IDIBELL-UB Research Group, Pharmacotherapy, Pharmacogenomics and Pharmaceutical Technology, Avinguda Granvia, 199-203, 08908 L’Hospitalet de Llobregat, Spain; 3 SOM Biotech S.L, Barcelona Scientific Park, C/Baldiri Reixac, 4, 08028 Barcelona, Spain; insa@sombiotech.com

**Keywords:** hydrophilic matrix tablets, tolcapone, sustained release, particle size, SeDeM diagram, direct compression

## Abstract

Hydrophilic matrix tablets are a type of sustained release dosage form characterized by distributing a drug in a matrix that is usually polymeric. Tolcapone is a drug that inhibits the enzyme catechol-*O*-methyl transferase. In recent years, it has been shown that tolcapone is a potent inhibitor of the amyloid aggregation process of the transthyretin protein, and acts by stabilizing the structure of the protein, reducing the progression of familial amyloid polyneuropathy. The main objective of this study was to obtain a sustained release tablet of tolcapone for oral administration with a preferred dosage regimen of 1 administration every 12 or 24 h and manufactured, preferably, by direct compression. The SeDeM Diagram method has been used for the formulation development of hydrophilic matrix tablets. Given the characteristics of tolcapone, the excipient selected for the formation of the polymeric matrix was a high viscosity hydroxypropylmethylcellulose (Methocel^®^ K100M CR). A decrease in the particle size of tolcapone resulted in a slower dissolution release of the formulation when the concentration of the polymer Methocel^®^ K100M CR was below 29%. These surprising and novel results have given rise to patent number WO/2018/019997.

## 1. Introduction

Modified release pharmaceutical forms are designed with the aim of modifying the rate or the site of release of a drug, with respect to the immediate release pharmaceutical forms of the same active ingredient [1].

Sustained-release or extended-release forms are types of modified-release dosage forms characterized by an initial release of the drug in enough quantities to produce therapeutic action or even a small excess. This excess is never harmful to the organism but it allows the slow release of the drug to continue, sometimes with a speed that is not always equal to the speed of elimination [2].

Matrix tablets are a type of sustained-release dosage. The drug is distributed in a matrix that is usually polymeric. This matrix hinders the access of the solution medium to the surface of the particles and make the drug diffusion towards the outside matrix difficult [3].

Hydrophilic matrix tablets of unlimited swelling are tablets in which the active ingredient is mainly released through a diffusion process and, to a lesser extent, through an erosion process. In the diffusion process, the tablets, on contact with water, quickly form a gel on their entire surface. This gel establishes a diffusion barrier for active ingredient molecules that enter a state of dissolution. As the polymeric excipient constituting the matrix is hydrated, gelation proceeds at some speed towards the solid core, where the polymer is in a non-hydrated state. In the erosion mechanism, the external gelled layer, when eroded, also contributes to the process of sustained release of the active ingredient [3].

In the manufacture of hydrophilic matrices of unlimited swelling, hydrophilic polymers, mainly cellulosic derivatives, are used. One of the most used polymers is hydroxypropylmethylcellulose (HPMC). HPMC is a hydrophilic cellulose derivative with non-ionic, inert, odorless fillers and is obtained from purified cellulose [4]. Hydroxypropylmethylcellulose is one of the most popular hydrophilic polymers for the formulation of controlled drug release systems since 1960 [5,6,7]. Among its most important characteristics are its high gelling capacity and swelling, which in turn have a significant effect on the release kinetics of the drug incorporated into the system. Furthermore, it is easy to compress and has the ability to house a large number and diversity of drugs [8,9,10]. It has also been found that this polymer does not influence the variables of the manufacturing process of matrix tablets for drug release [11,12,13].

Tolcapone is a drug that inhibits the enzyme catechol-*O*-methyl transferase (COMT) [14]. It is currently used in the treatment of Parkinson’s disease as a supplement to the medication Levodopa/Carbidopa under the name of Tasmar^®^. It is marketed in immediate release tablets manufactured by wet granulation prior to compression and final coating [15]. In recent years, it has been shown that tolcapone is a potent inhibitor of the amyloid aggregation process of the transthyretin protein, and acts through stabilizing the structure of the protein, reducing the progression of a disease called familial amyloid polyneuropathy [16]. This new indication has been patented by SOM INNOVATION BIOTECH, S.L with the reference number WO2013060668 A1 [17].

This drug has been designated as an orphan drug by the US Food and Drug Administration for the treatment of transthyretin amyloidosis (the most common form of familial amyloidosis). Patients with familial amyloid polyneuropathy are chronic, polymedicated patients with a deficient quality of life [18]. Any improvement in the treatment, both at the level of administration route and the dosage schedule of the medications, can result in an improvement in quality of life and a correct treatment compliance [19]. Sustained release pharmaceutical forms, due to their intrinsic characteristics, can help to minimize poor patient compliance, multiple dosing and see-saw fluctuations [20].

Tolcapone is rapidly absorbed, with a Tmax of approximately 2 h. Clinical trials have shown that with multiple dose administration, tolcapone demonstrated linear pharmacokinetics in the 50 to 400 mg range. However, during multiple dosing with 400 and 800 mg three times a day, there was some accumulation of the drug. With the design of a sustained release formulation, plasma levels could be maintained by avoiding overdoses and, at the same time, improving the patient’s quality of life [21].

The main objective of this study was to obtain a sustained release tablet of tolcapone for oral administration with a preferred dosage regimen of 1 administration every 12 or 24 h and manufactured, preferably, by direct compression. After studying the active ingredient and due to its intrinsic characteristics such as low compressibility and bad flowability, among others, we decided to develop a hydrophilic matrix to obtain the desired formulation. 

The SeDeM Diagram method has been used for the formulation of hydrophilic matrix tablets. This method is based on the determination of five parameters which allow us to predict the suitability of the powder (either API or excipient) or of a mixture to be compressed directly according to the good compression index (GCI) obtained [22]. It has been shown to be a suitable tool for pre-formulation and formulation due to its ability to characterize the galenic properties of excipients in order to define their suitability for direct compression [22,23,24,25,26,27]. Recently, some authors have validated the tool’s usefulness [28,29,30,31].

## 2. Materials and Methods

### 2.1. Materials

The active substance under study is tolcapone (batches: SOM0114599 and SOM0714600) (CCN Industries LTD via Porschem Pharm).

The excipients studied are Vivapur^®^ 102 (JRS, Rosenberg, Germany), Avicel^®^ PH 101 (FMC Corp, Brusseles, Belgium), Kleptose^®^ (ROQUETTE, Roquette Frères, Lestrem, France), Kollidon^®^ VA 64 (BASF, Ludwigshafen, Germany), Prosolv^®^ HD90 (JRS, Rosenberg, Germany), Isomalt^®^ 721 (GalenIQ, Manheim, Germany), Methocel^®^ K100M CR (Colorcon, Dartford, UK), talc (Fagron, Terrassa, Spain), magnesium stearate (Fagron, Terrassa, Spain), and colloidal silicon dioxide (Fagron, Terrassa, Spain).

### 2.2. Methods

#### 2.2.1. API Characterization

The SeDeM Method has been used to assess the suitability of an active ingredient for its direct compression. The parameters considered in the SeDeM Method are the following:

- Bulk density (Da)
- Tapped density (Dc)
- Inter-particle porosity (I_e_)
- Carr index (IC)
- Cohesion index (Icd)
- Hausner ratio (IH)
- Angle of repose (α)
- Powder flow (t”)
- Loss on drying (%HR)
- Hygroscopicity (%H)
- Particle size (%Pf)
- Homogeneity index (I_θ_)

These tests are grouped into five factors based on the physical characteristics of the powder and the functionality of the drug:

(1) Dimensional Parameter: bulk density (Da) and tapped density (Dc).

These affect the size of the tablet and its capacity to pile up. In addition, these tests are used in the calculation of other mathematical indexes for determination of the compressibility parameter.

(2) Compressibility Parameter: inter-particle porosity (Ie), Carr index (IC) and cohesion index (Icd).

These affect the compressibility of the powder.

(3) Flowability/Powder Flow Parameter: Hausner ratio (IH), angle of repose (α) and flowability (t”).

These influence the flowability of the powdered substance when compressed.

(4) Lubricity/Stability Parameter: loss on drying (%HR) and hygroscopicity (%H).

These affect the lubricity and future stability of the tablets.

(5) Lubricity/Dosage Parameter: %particles < 50 mcm and homogeneity index (I_θ_).

These influence the lubricity and dosage of the tablets.

These parameters are determined by means of the new SeDeM Diagram method, based on known equations [22,23], and which have been duly validated in reproducible experimental tests, as shown in Table 1.

The methods used for each test are described below [22]:

- Bulk density (Da): The method is described in Section 2.9.34 of the European Pharmacopeia (European Pharmacopeia 9th edition, 2017).
- Tapped density (Dc): The volume taken is the value obtained after 2500 strokes using a settling apparatus with a graduated cylinder (voluminometer).
- Inter-particle porosity (Ie) of the powder mixture is calculated from the following equation: Ie = Dc − Da/Dc × Da.
- Carr index (IC%) [7,8,9]: This is calculated from Da and Dc as: IC = (Dc − Da/Dc) × 100.
- Cohesion index (Icd): This index is determined by compressing the powder, preferably in an eccentric press. First of all, the mean hardness (N) of the tablets is calculated, the raw powder is tested, but if it cannot be compressed, 3.5% of the following mixture is added to the mix: talc 2.36%, Aerosil ^®^ 200 0.14% and magnesium stearate 1.00%.
- Hausner ratio (IH) (European Pharmacopeia 9th edition, 2017): This method is described in Section 2.9.34 of the European Pharmacopeia (European Pharmacopeia 9th edition, 2017). This is calculated from Da and Dc as: IH = Dc/Da.
- Angle of repose (α): The method is described in Section 2.9.36 of the European Pharmacopeia (European Pharmacopeia 9th edition, 2017). This is the angle of the cone formed when the product is passed through a funnel with the following dimensions: height 9.5 cm, upper diameter of spout 7.2 cm, internal diameter at the bottom, narrow end of spout 1.8 cm. The funnel is placed on a support 20 cm above the table surface, centered over a millimeter-grid sheet on which two intersecting lines are drawn, crossing at the centre. The spout is plugged and the funnel is filled with the product until it is flush with the top end of the spout when smoothed with a spatula. Remove the plug and allow the powder to fall onto the millimetre sheet. Measure the four radii of the cone base with a slide caliper and calculate the mean value (r). Measure the cone height (h). Deduce α from tan (α) = h/r.
- Flowability (t”): The method is described in Section 2.9.16 of the European Pharmacopeia (European Pharmacopeia 9th edition, 2017). It is expressed in seconds and tenths of a second per 100 g of sample, with a mean value of three measurements.
- Loss on drying (%HR): This is measured by the method described in Section 2.2.32 in the European Pharmacopeia (European Pharmacopeia 9th edition, 2017). The sample is dried in an oven at 105 °C ± 2 °C, until a constant weight is obtained.
- Hygroscopicity (%H): Determination of the percentage increase in sample weight after being kept in a humidifier at a relative humidity of 76% (±2%) and a temperature of 22 °C ± 2 °C for 24 h.
- Percentage of particles measuring < 50 μm (%Pf): Particle size is determined by means of the sieve test following the general method 2.9.12 of the European Pharmacopeia (European Pharmacopeia 9th edition, 2017). The value returned is the % of particles that pass through a 0.05-mm sieve when vibrated for 10 min at speed 10 (CISA^®^ vibrator).
- Homogeneity index (I_θ_): This is calculated according to the General method 2.9.12 of the European Pharmacopeia (European Pharmacopeia 9th edition, 2017).

To determine particle size by means of the sieve test, the grain size of a 100 g sample is measured by subjecting a sieve stack to vibration for 10 min at speed 10 (CISA^®^ vibrator). The sieve sizes used are 0.355 mm, 0.212 mm, 0.100 mm and 0.05 mm. The percentage of product retained in each sieve is calculated and the amount that passes through the 0.05 mm sieve is measured. The percentage of fine particles (<50 μm) (%Pf) was calculated as described above. Note that if this percentage is higher than that calculated in the complete sieve test, it is because some of the particles adhere to the product retained in the sieves during the grain-size test, and the percentage of <50 μm particles found may be lower than the true figure. The following equation is then applied to the data obtained.

Equation (1) named in Table 1 is:

(1) $$I_{\theta }=\frac{F_{m}}{100+\left(d_{m}{-d}_{m-1}\right)F_{m-}{}_{1}+\left(d_{m+1}{-d}_{m}\right)F_{m+1}+\left(d_{m}{-d}_{m-2}\right)F_{m-2}+\ldots \;+\;\left(d_{m}{-d}_{m-n}\right)F_{m-n}+\left(d_{m+n}{-d}_{m}\right)F_{m+n}}$$

where I_θ_: relative homogeneity index, particle-size homogeneity in the range of the fractions under study.

F_m_: Percentage of particles in the majority range.

F_m−1_: Percentage of particles in the range immediately below the majority range.

F_m+1_: Percentage of particles in the range immediately above the majority range.

n: Order number of the fraction under study, within a series, with respect to the majority fraction.

d_m_: Mean diameter of the particles in the majority fraction.

d_m−1_: Mean diameter of the particles in the fraction of the range immediately below the majority range.

d_m+1_: Mean diameter of the particles in the fraction of the range immediately above the majority range.

Once the values had been obtained following the specific methods, certain limits were set, based on the study of the chosen parameters and the values described in the handbook of pharmaceutical excipients [4]. The next step was to convert the numeric limits for each SeDeM Diagram parameter to radius values (r), in accordance with Table 2 [23,32,33,34]. 

When all radius values were 10, the SeDeM Diagram takes the form of a circumscribed regular polygon, drawn by connecting the radius values with linear segments. The results obtained from the earlier parameter calculations and conversions are represented by the radius. The figure formed indicates the characteristics of the product and of each of the parameters that determine whether or not the product is suitable for direct compression. In this case, the SeDeM Diagram is made up of 12 parameters, which would form an irregular 12-sided polygon. 

To determine whether or not the product is acceptable for direct compression in numerical form, the following indexes were calculated based on the SeDeM Diagram as:

Parameter index (IP) = No × p ≥ 5/No × Pt

No × p ≥ 5: Indicates the number of parameters whose value is equal to or higher than 5.

No × Pt: Indicates the total number of parameters studied.

Parameter profile index (IPP) IPP = mean r ≥ 5 of all parameters

Mean r = mean value of the parameters calculated.

The acceptability limit would correspond to: IPP = mean r ≥ 5

Good compression index GCI = IPP × f

where f is the reliability factor and is calculated as follows:

f = polygon area/circle area

The acceptability limit was calculated by:

GCI = IPP × f > 5

Particle size determination was performed following the general method 2.9.31 European Pharmacopoeia with a particle size laser analyzer MASTERSIZER 2000 (Malvern) featuring a wet dispersion unit HYDRO 2000 SM for small volumes of sample. The following conditions were met:

- Material:

- Sample: tolcapone suspension (500 mg of tolcapone in 50 mL of water for injection)
- Refraction index: 1.59 (default)
- Dispersant: water for injection
- Refraction index: 1.33

- Cycles:

- Measurements for aliquot: 3
- Lag time: 12 s
- Pump stirring velocity: 2500 rpm

- Measuring time:

- Measure: 12 s (in triplicates)
- Measure snaps: 12,000
- Background: 10 s
- Background snaps: 10,000

Three readings have been performed for each measure.

#### 2.2.2. Preparation of the Different Formulations

A formulation is designed to obtain sustained release hydrophilic matrix tablets which can be obtained by direct compression. Different diluents of direct compression were chosen applying the SeDeM Diagram method for its characterization [35].

Given the pharmacokinetic characteristics of tolcapone (rapid absorption, with a Tmax of approximately 2 h), the excipient selected for the formation of the polymeric matrix was hydroxypropylmethylcellulose, an excipient widely used in this kind of formulation.

As it is well known, there are different types of hydroxypropylmethylcellulose. In this study, a high viscosity polymer (Methocel^®^ K100M CR) was chosen because of the need to obtain a matrix that establishes a sufficiently compact gelation barrier to allow a slow diffusion of the drug.

Six diluents (Vivapur^®^ 102, Avicel^®^ PH101, Kleptose^®^ among others) were characterized using the SeDeM method. Depending on the API characterization results, the direct compression diluent will be chosen. The diluent provides the bulk of the tablet and is also responsible for flow and compaction properties.

Anhydrous colloidal silica was chosen as a glidant due to its properties in improving the flowability of the mixer. Two lubricants, magnesium stearate and talc, are added to the formulation to enhance the lubricity and flow properties of the formulation. Fixed concentrations of these last three components (anhydrous colloidal silica 0.30%, magnesium stearate 0.05% and talc 0.12%) as well as tolcapone (300 mg per tablet) were established, following previous work [22,24].

The concentrations of the polymer (hydroxypropylmethylcellulose) and the diluent chosen are modified depending on the difficulties that may arise during the manufacturing and the results obtained in the dissolution test.

The manufacturing process of the formulations is as follows: the raw materials are weighed individually into polyethylene bags. Then they are sieved in a 0.8 mm sieve, transferred to a suitable container, and mixed together (API and all the excipients, included magnesium stearate) for 20 min at 20 rpm in the Glatt biconical mixer. 

#### 2.2.3. Tablets Preparation

The blends were compressed in a Bonals^®^ (Cornellà de Ll., Spain) continuous eccentric press, provided with 19 mm × 10 mm punches. 

#### 2.2.4. Tablets Characterization

In the characterization of the tablets the method applied was the dissolution test. The dissolution test was performed following USP38 monograph of tolcapone’s tablet. This monograph is described for immediate release tablet, but in this case the measuring time is extended for 24 h.

The sample was read directly using 1 mm cuvettes in a Dissolution Apparatus Type 2 (Agilent Technologies 708-DS). The volume of the media (pH 6.8 phosphate buffer solution containing 1% of sodium lauryl sulphate) was 900 mL at a temperature between 36.5 and 37.5 °C using a paddle for stirring at a speed of 75 rpm. The analysis of the sample was carried out by autosampling, followed by filtration, spectrophotometric (Agilent Technologies 8453) reading and return of the solution to the vessel. The measurement was done a wavelength of 271 nm.

The measuring time was carried out at start of the assay, 5, 10, 15, 30, 45, 60, 90, 105, 120, 150, 180, 210, 240, 270, 300, 330, 360, 390, 420, 450, 480, 510, 540, 570, 600, 630, 660, 690, 720, 750, 780, 810, 840, 870, 900, 930 until 1440 min.

The dissolution profile fit with the objective of dissolving 80% of the active ingredient in 8 h from the start of the trial, which according to USP38 specifications established for sustained release dosage forms monographs, corresponds to a suitable release for once daily administration.

## 3. Results and Discussion

### 3.1. Results for the API Characterization

**Figure 1:**
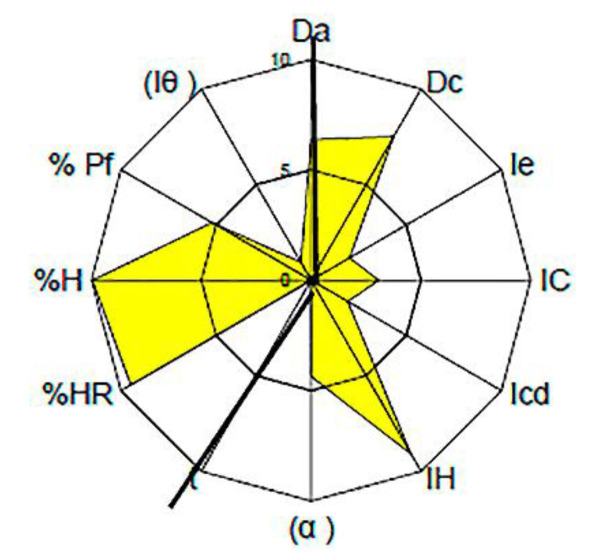
SeDeM Diagram of tolcapone batch SOM0114599. Da: bulk density; Dc: tapped density; Ie: inter-particle porosity; IC: Carr index; Icd: cohesion index; IH: Hausner ratio; α: angle of repose; t”: powder flow; %HR: loss on drying; %H: hygroscopicity; %Pf: particle size; I_θ_: homogeneity index.

**Figure 2:**
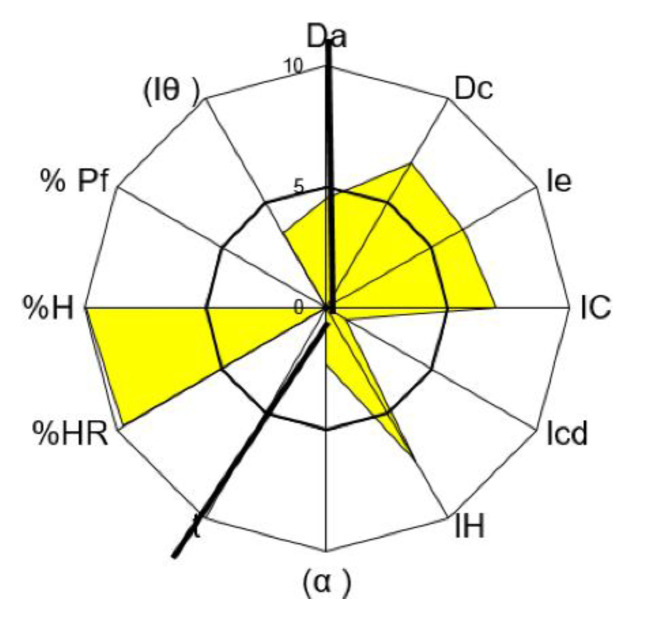
SeDeM Diagram of tolcapone batch SOM0714600. Da: bulk density; Dc: tapped density; Ie: inter-particle porosity; IC: Carr index; Icd: cohesion index; IH: Hausner ratio; α: angle of repose; t”: powder flow; %HR: loss on drying; %H: hygroscopicity; %Pf: particle size; I_θ_: homogeneity index.

For tolcapone batch SOM0114599, the obtained value for the parameter index was 0.50, for parameter profile index it was 5.00 and for the good compression index it was 4.76. For batch SOM0714600 the obtained value for the parameter index was 0.50, for the parameter profile index it was 4.89 and for the good compression index it was 4.66. All these values indicate that tolcapone is amenable for the preparation of tablets by direct compression (Figure 1 and Figure 2) [23].

In both batches, the average values in the “Dimensions” and “Lubricity/Stability” incidences were higher than 5. These are very acceptable results for the compression of the substance.

The average value for “Compressibility” incidence was lower than 5, with a cohesion index smaller than 2. These low values show a potential difficulty of tolcapone to be compressed in the absence of excipients. These results suggest that appropriate excipients may be necessary to increase compressibility. The average value for “Flowability/Powder flow” incidence was lower than 4.5. It should be noted that the powder flow assay has not been performed because the flow capacity was 0 due to the powder not passing through the funnel. Addition of appropriate lubricants must be considered in order to achieve a formulation with correct compression properties, as has been mentioned before. The average value of “Lubricity/Dosage” incidence was lower or equal to 3, with a homogeneity index lower than 4. These values are also low, confirming the conclusions obtained from the analysis of the previous parameters: the active substance presents rheological characteristics which must be corrected with an appropriate formulation.

Regarding the particle size, there were substantial differences between the two batches. As indicated in Table 3, the SOM0714600 batch had a particle size of up to 80% lower than the SOM0114599 batch.

### 3.2. Results for the Final Formulations

First of all, the six diluent excipients chosen were characterized following the methodology of SeDeM Diagram with the objective of choosing the excipient with the capacity of correcting the deficient parameters of tolcapone.

After the evaluation of the results obtained from the characterization of the six diluents (Table 4), Vivapur^®^ 102 (microcrystalline cellulose) was chosen as the suitable excipient to correct the deficiencies of this API batch for its galenical characteristics (compressibility mean incidence of 8.91 and flowability/powder flow mean incidence of 4.00) [24].

The choice of this diluent was made taking into account that the compressibility value of the API is extremely low, and it is necessary to increase it significantly in order to perform a direct compression. Thus, although the value of flowability is not very high, it should be noted that this would be increased with the addition of non-stick and lubricating excipients. So, due to its intrinsic characteristics, Vivapur^®^ 102 is able to compensate for the deficit cohesiveness of tolcapone.

Initially, in previous studies of preformulation, the feasibility of obtaining mixtures of microcrystalline cellulose and hydroxypropylmethylcellulose with the possibility of being compressed by direct compression was verified.

The experimental design used is the design of mixtures (References 1 to 6), wherein the polymer concentration ranges between 20% (*w*/*w*) and 35% (*w*/*w*). The diluent concentration is modified in function of polymer concentration.

Fixed concentrations of colloidal silicon dioxide, magnesium stearate and talc as well as tolcapone (300 mg per tablet) were established.

The different formulations are shown in Table 5. 

**Figure 3:**
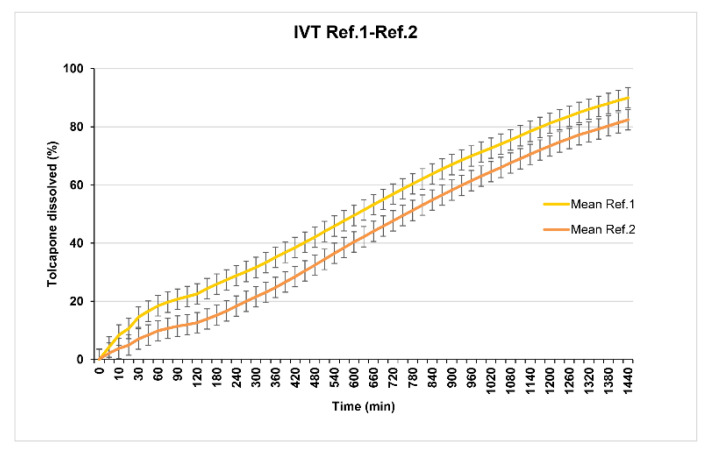
References 1 and 2 dissolution test results.

Reference 1 (29% of Methocel^®^ K100M CR) and Reference 2 (35% of Methocel^®^ K100M CR) present in vitro release profiles that are too slow (Figure 3).

Taking into account that the objective is to obtain a formulation that should be administered, if possible once a day, analyzing both profiles it was observed that neither of them release 80% of tolcapone at 480 min (8 h) according to the USP38 specifications established for sustained release dosage forms monographs. For this reason, the release was considered too slow and the percentage of polymer was reduced.

**Figure 4:**
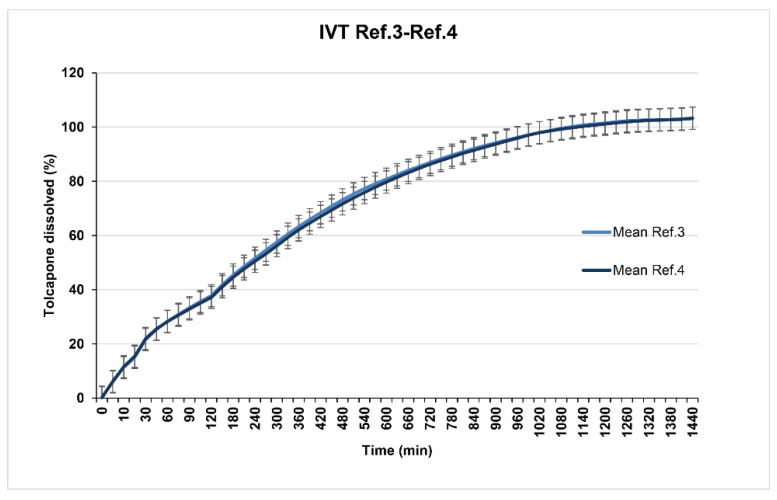
References 3 and 4 dissolution test results.

**Figure 5:**
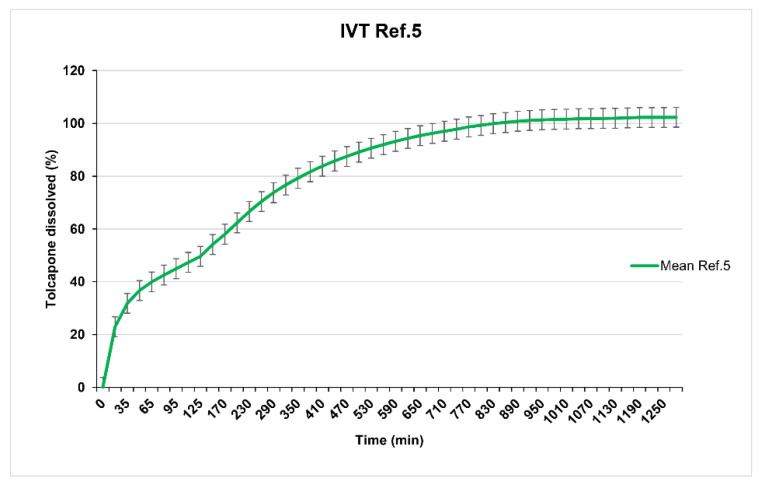
Reference 5 dissolution test results.

Reference 3 (23% of Methocel^®^ K100M CR) and Reference 4 (26% of Methocel^®^ K100M CR) show in vitro release profiles (overlapped as seen in Figure 4) adjusted to the established objective since at 8 h, 70% of tolcapone had been released. Reference 5 (20% of Methocel^®^ K100M CR) presented a suitable in vitro release profile (Figure 5) with 80% of tolcapone released at 8 h as intended.

This is a drug release probably produced by a mixed mechanism of diffusion and erosion, which would explain the shape of the dissolution profile: initially fast in the first 35 min (the erosion mechanism predominates), and then to a sigmoid profile as dissemination intervenes to a greater extent. This is due to the lower proportion of Methocel K110M CR that exists in the tablet with respect to the other formulations. The lower percentage of this excipient means that there is no such slow release mediated by a predominant diffusion mechanism and the irregularity caused by the mixed drug release mechanism is manifested more clearly, with more erosion mechanism intervention.

As shown by the studies conducted by Guojie Xu and Hisakazu [36], it is confirmed with tolcapone’s formulations that the higher the polymer concentrations are, the slower the in vitro release profile of the drug is.

Reference 6 (with the same concentration of hydroxypropylmethylcellulose as Reference 5, 20%) is manufactured to study the impact that can represent the change of physicochemical characteristics (smaller particle size) of tolcapone (batch SOM0714600).

**Figure 6:**
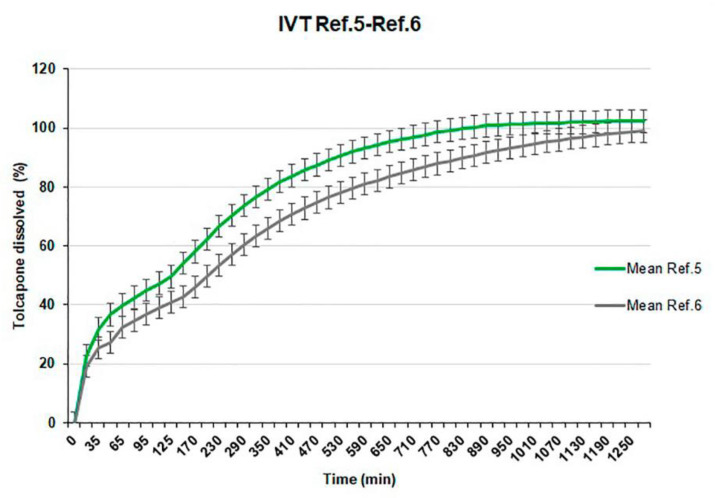
Comparative dissolution test results of References 5 and 6.

The manufacturing process of this reference presents no additional difficulty but the in vitro release profile is surprisingly slower than the profile obtained for Reference 5, manufactured with the initial batch of tolcapone (batch SOM0114599) (Figure 6).

To demonstrate this, a statistical study was carried out. The data obtained were processed by a simple regression method adjusting these data to a potential equation [Y(%dissolved) = A·X(time)^B^] which was optimal to explain the evolution of the dissolution process within a bounded time interval (50 to 650 min). 

The results obtained are as follows: 

Reference 5: 

Adjusted equation: Y = 2.0656·X^0.3887^

Regression analysis–Multiplicative model: Y = a*X^b

Dependent variable: (Comp5)

Independent variable: (Time5)

Selection variable: Time5 > 50 & Time5 < 650.
**Standard Statistical****Parameter****Estimation****Error****T*****p*-Value**Ordinate2.065570.028958271.32950.0000Slope0.3887120.0051641175.27190.0000**Analysis of Variance****Source****Square sum****GL****Average squar****F-Ratio*****p*-Value**Model1.6731811.673185665.860.0000Residue0.00590618200.000295309

Total (Corr.) 1.67909 21; Correlation coefficient = 0.99824; Square-R = 99.6483 percentage.

From the results of the analysis of variance (ANOVA) of the adjusted model, it is observed that they explain 99.65% of the quadratic variation of dissolution as a function of time, resulting in a correlation coefficient r = 0.9982 between the observed values and values adjusted with the help of the equation. Thus, it may be concluded that the dissolution kinetic of the product of Reference 5 fits a mathematical model of simple potential regression with a coefficient A = 2.0656 and a power B = 0.3887. 

Reference 6: 

Adjusted equation: Y = 1.6295·X^0.4347^

Regression analysis–Multiplicative model: Y = a*X^b

Dependent variable: (Comp6)

Independent variable: (Time6)

Selection variable: Time6 > 50 & Time6 < 650
**Standard Statistical****Parameter****Estimation****Error****T*****p*-Value**Ordinate1.629450.028756.77530.0000Slope0.4347350.0051309684.72790.0000**Analysis of Variance****Source****Square sum****GL****Average squar****F-Ratio*****p*-Value**Model1.9502611.950267178.810.0000Residue0.00543337200.000271669

Total (Corr.) 1.95569 21; Correlation coefficient = 0.99861; Square-R = 99.7222 percentage.

From the results of the analysis of variance (ANOVA) of the adjusted model it is observed that they explain 99.72% of the quadratic variation of dissolution as a function of time, resulting in a correlation coefficient r = 0.9986 between the observed values and values adjusted with the help of the equation. Thus, it may be concluded that the dissolution kinetic of Reference 6 fits a mathematical model of simple potential regression with a coefficient A = 1.6295 and a power B = 0.4347.

Comparison between the dissolution profiles of the compositions of Reference 5 and Reference 6 were adjusted to the potential linear regression.

A comparative statistical study was carried out based on confidence intervals at 95% for each experimental value to determine whether there exist statistically significant differences between the kinetics of dissolution of the formulation of Reference 5 and Reference 6 within the time interval of 50 min to 650 min. Insofar as these intervals do not overlap each other (which can be seen when plotting said prediction intervals), it can be concluded that there are significant differences between the kinetics of dissolution obtained for each of the formulations. 

**Figure 7:**
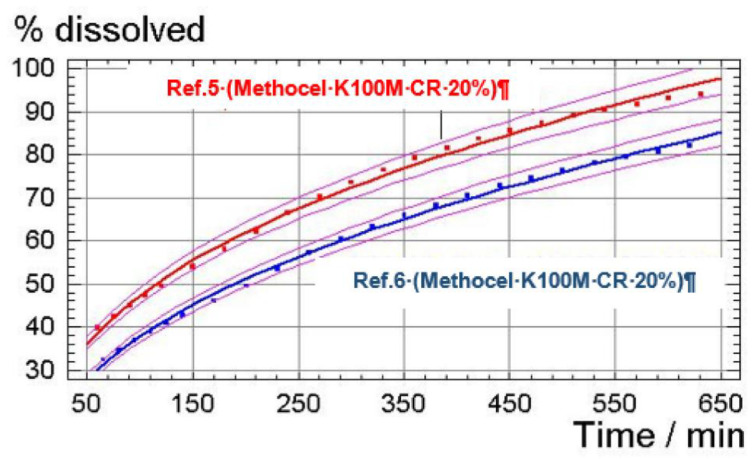
Representation of experimental values and the adjusted models and prediction intervals for a confidence interval of 95%.

The representation of the experimental values and the adjusted models and prediction intervals for a confidence interval of 95% is presented in Figure 7.

It is noted that for the entire experimental range studied, the adjusted dissolution values for the composition of Reference 5 are, with a confidence interval of 95%, higher than those for the composition of Reference 6. Thus, it may be concluded that the composition of Reference 5 (comprising non-micronized tolcapone) shows a higher dissolution rate than that of the composition of Reference 6 (comprising micronized tolcapone). 

These results are surprising since one would expect the release of Reference 5 to be slower than that of Reference 6 due to the lower particle size of the active ingredient in the last reference.

The dissolution rate of any drug is conditioned by a series of parameters, which are related in the Noyes–Whitney equation [37],

dC/dt = K·A·(Cs − C)

where dC/dt is the dissolution rate, K is the velocity constant that describes the diffusion of the drug to the medium (dissolution constant), A is the surface of the solid to dissolve (directly related to the particle size of the solid), Cs is the saturation concentration in the liquid medium surrounding the solid to be dissolved and C is the concentration in the solvent at a given time.

As shown in the Noyes–Whitney equation, the dissolution rate depends on the physicochemical characteristics of the active ingredient, in addition to the formulation and solvent characteristics. Some of these characteristics are the coefficient of solubility of each defined chemical substance, the crystalline morphology (polymorphism) and the particle size [38].

The particle size influences the specific surface of the particle so, according to the Noyes–Whitney equation, a smaller particle size exponentially increases the dissolution rate (smaller particle size, greater contact specific surface with the dissolution medium).

Likewise, if, through the formulation of the drug, an increase of the solubility coefficient of the active ingredient is achieved, the value of the solution constant K of the Noyes–Whitney equation will increase and, consequently, the rate of dissolution will increase too.

In short, when the solubility of the drug is a problem in achieving a suitable bioavailability and the limitation is precisely in the dissolution of it, procedures aimed at improving the dissolution rate are required [39], such as the reduction of particle size, the formation of drug–excipient complexes or the formation of solid dispersions, among others that increase the solubility coefficient of the drug.

Thus, the results obtained are surprising and novel and have given rise to the patent number WO2018019997 [40].

## 4. Conclusions

The research carried out has made it possible to obtain, using the SeDeM Diagram tool, a hydrophilic-matrix-sustained release tablet of tolcapone for the treatment of familial amyloid polyneuropathy. Reference 5 is proposed as the definitive prototype formula because it presents appropriate characteristics for direct compression and dissolution.

A decrease in the particle size of tolcapone results in a slower dissolution release of the formulation when the concentration of the polymer Methocel^®^ K100M CR is below 29%. In this case, tolcapone, a substance insoluble in water, forms a dispersion in the gelled matrix more viscous as the particle size of tolcapone becomes smaller, which would cause a slower release of the active substance.

## 5. Patents

These results have given rise to patent number WO/2018/019997.

## Figures and Tables

**Table 1:** Parameters and equations used in SeDeM methodology.

Incidence	Parameter	Symbol	Unit	Equation
Dimension	Bulk density	Da	g/mL	D = P/Va
	Tapped density	Dc	g/mL	Dc = P/Vc
Compressibility	Inter-particle porosity	Ie	_	IE = Dc − Da/Dc × Da
	Carr Index	IC	%	IC = (Dc − Da/Dc) × 100
	Cohesion index ^a^	Icd	N	Experimental
Flowability/Powder flow	Hausner ratio	IH	_	IH = Dc/Da
	Angle of repose	(α)	_	Tgα = h/r
	Powder flow	t”	s	Experimental
Lubricity/Stability	Loss on drying	%HR	%	Experimental
	Hygroscopicity	%H	%	Experimental
Lubricity/Dosage	Particles < 50 μm	%PF	%	Experimental
	Homogeneity index ^b^	(I_θ_)	_	Equation (1)

^a^ Hardness (N) of the table obtained with the product in question, alone or blended with lubricants if highly abrasive. ^b^ Determines particle size. In accordance with the percentages of the different particle-size fractions by applying Equation (1).

**Table 2:** Conversion of limits for each parameter into radius values (r). The twelve parameters are represented in a 12-sided polygon.

Incidence	Parameter	Limit Value	Radius (r)	Factor Applied to v
Dimension	Bulk density	0–1 g/mL	0–10	10v
	Tapped density	0–1 g/mL	0–10	10v
Compressibility	Inter-particle porosity	0–1.2	0–10	10v/1.2
	Carr Index	0–50 (%)	0–10	v/5
	Cohesion index	0–200 (N)	0–10	v/290
Flowability/Powder flow	Hausner ratio	1–3	10–0	5(3 − v)
	Angle of repose	50–0 (°)	0–10	10 − (v/5)
	Powder flow	20–0 (s)	0–10	10 − (v/2)
Lubricity/Stability	Loss on drying	0–10 (%)	10–0	10 − v
	Hygroscopicity	20–0 (%)	0–10	10 − (v/2)
Lubricity/Dosage	Particles < 50 μm	50–0 (%)	0–10	10 − (v/5)
	Homogeneity index	0–2 × 10^−2^	0–10	500v

**Table 3:** % decrease in particle size of batch SOM0714600 with respect to batch SOM0114599.

Particle Volume	Maximum Particle Size Batch SOM0114599	Maximum Particle Size Batch SOM0714600	% Decrease in Particle Size of Batch SOM0714600 with Respect to Batch SOM0114599
10%	22,449 µm	6085 µm	72.89%
50%	87,223 µm	17,620 µm	79.79%
90%	30,789 µm	54,329 µm	82.35%
100%	1,096,478 µm	316,228 µm	71.15%

**Table 4:** Individual radius parameters, mean incidences and parametric index for diluents excipients.

EXCIPIENT	Parameters ( r )												Mean Incidence				Index			
	Da	Dc	I_e_	IC	Icd	IH	(a)	t”	%HR	%H	%pf	(I_θ_)	Dimension	Compressibility	Flowability/Powder Flow	Lubricity/Stability	Lubricity/Dosage	IP	IPP	GCI
Vivapur^®^ 102	3.32	5.28	9.32	7.42	10.00	7.05	3.44	1.50	5.46	7.59	3.46	3.05	4.30	8.91	4.00	6.53	3.26	0.58	5.57	5.31
Avicel^®^ PH101	3.47	4.63	6.02	5.01	10.00	5.55	3.46	0.00	3.84	8.17	3.38	10.00	4.05	7.01	3.01	6.01	6.69	0.50	5.29	5.04
Kleptose^®^	5.58	8.46	5.08	6.81	10.00	4.95	3.51	6.50	0.00	8.12	3.60	1.90	7.02	7.30	4.98	4.06	2.75	0.58	5.38	5.12
Kollidon^®^ VA 64	2.53	3.43	8.64	5.25	6.91	5.48	6.04	5.25	3.19	2.85	8.40	5.50	2.98	6.93	5.59	3.02	6.95	0.67	5.29	5.03
Prosolv^®^ HD90	4.86	5.96	3.17	3.69	10.00	5.91	5.99	6.75	3.44	8.86	6.24	10.00	5.41	5.62	6.22	6.15	8.12	0.67	6.24	5.94
Isomalt^®^ 721	4.40	5.60	4.06	4.29	10.00	5.76	6.24	6.85	4.01	9.89	9.00	2.00	5.00	6.11	6.28	6.95	5.50	0.58	6.01	5.72

Da: bulk density; Dc: tapped density; I_e_: inter-particle porosity; IC: Carr index; Icd: cohesion index; IH: Hausner ratio; α: angle of repose; t”: powder flow; %HR: loss on drying; %H: hygroscopicity; %Pf: particle size; I_θ_: homogeneity index.

**Table 5:** Composition of the different formulations (refs.1–6).

Components	Ref.1	Ref.2	Ref.3	Ref.4	Ref.5	Ref.6
	%	%	%	%	%	%
Tolcapone	37.50%	37.50%	37.5%	37.50%	37.5%	37.5%
Methocel^®^ K100M CR	29.00%	35.00%	23.00%	26.00%	20.00%	20.00%
Vivapur^®^ 102	33.03%	27.03%	39.03%	36.03%	42.03%	42.03%
Talc	0.12%	0.12%	0.12%	0.12%	0.12%	0.12%
Magnesium stearate	0.05%	0.05%	0.05%	0.05%	0.05%	0.05%
Colloidal silicon dioxide	0.30%	0.30%	0.30%	0.30%	0.30%	0.30%
